# HIV infection of non-classical cells in the brain

**DOI:** 10.1186/s12977-023-00616-9

**Published:** 2023-01-13

**Authors:** Angela Wahl, Lena Al-Harthi

**Affiliations:** 1 International Center for the Advancement of Translational Science, University of North Carolina at Chapel Hill, Chapel Hill, NC USA; 2 Division of Infectious Diseases, Department of Medicine, University of North Carolina at Chapel Hill, Chapel Hill, NC USA; 3 Center for AIDS Research, University of North Carolina at Chapel Hill, Chapel Hill, NC USA; 4 Department of Microbial Pathogens and Immunity, Rush University Medical Center, Chicago, IL USA

**Keywords:** HIV, CNS, Brain, Astrocyte, Microglia, Non-classical cells, Neuroinvasion, Latency

## Abstract

HIV-associated neurological disorders (HAND) affect up to 50% of people living with HIV (PLWH), even in the era of combination antiretroviral therapy (cART). HIV-DNA can be detected in the cerebral spinal fluid (CSF) of approximately half of aviremic ART-suppressed PLWH and its presence is associated with poorer neurocognitive performance. HIV DNA + and HIV RNA + cells have also been observed in postmortem brain tissue of individuals with sustained cART suppression. In this review, we provide an overview of how HIV invades the brain and HIV infection of resident brain glial cells (astrocytes and microglia). We also discuss the role of resident glial cells in persistent neuroinflammation and HAND in PLWH and their potential contribution to the HIV reservoir. HIV eradication strategies that target persistently infected glia cells will likely be needed to achieve HIV cure.

## Background

HIV-associated neurocognitive disorders (HAND) is observed in approximately half of people living with HIV (PLWH) since the beginning of the HIV/AIDS epidemic [1–3]. HAND encompass a spectrum of cognitive impairments that cannot be attributed to co-infections or other causes. They are classified based on severity as asymptomatic neurocognitive impairment (ANI), mild neurocognitive disorder (MND), and HIV-associated dementia (HAD) [4]. In the pre-cART era, HIV associated dementia (HAD), the most severe form of HAND, was associated with advanced HIV infection and characterized by a progressive cognitive impairment, often accompanied by motor symptoms and behavioral changes, resulting in death within months [5–7]. The prevalence of HAD has dramatically declined in the cART era from ~ 15% to 2% [2, 8–10]. In contrast, the incidence of ANI and MND in PLWH has not declined despite widespread access to highly suppressive cART [2, 8, 10].

The persistence of HAND has been attributed to multiple factors including early CD4+ T cell depletion and neuronal damage prior to cART initiation, antiretroviral neurotoxicity, cardiovascular disease, and CNS inflammation [1, 3, 11]. Potential factors that contribute to sustained CNS inflammation in cART treated individuals include circulating microbial products and byproducts derived from the intestinal microbiome, conversion of proteasomes to immunoproteasomes in CNS cells, and the production of viral proteins such as gp120, Tat, and Vpr which promote CNS inflammation and neuronal damage, suggesting the persistence of HIV-infected cells in the CNS [3, 11–16]. In this review, we will address non-classical HIV infection of resident glia (astrocytes and microglia), their role as a reservoir for HIV and their contribution to persistent neuroinflammation, which together complicate cure strategies that must address the role of the brain as an HIV reservoir.

## Maintext

### HIV neuroinvasion

**Fig. 1:**
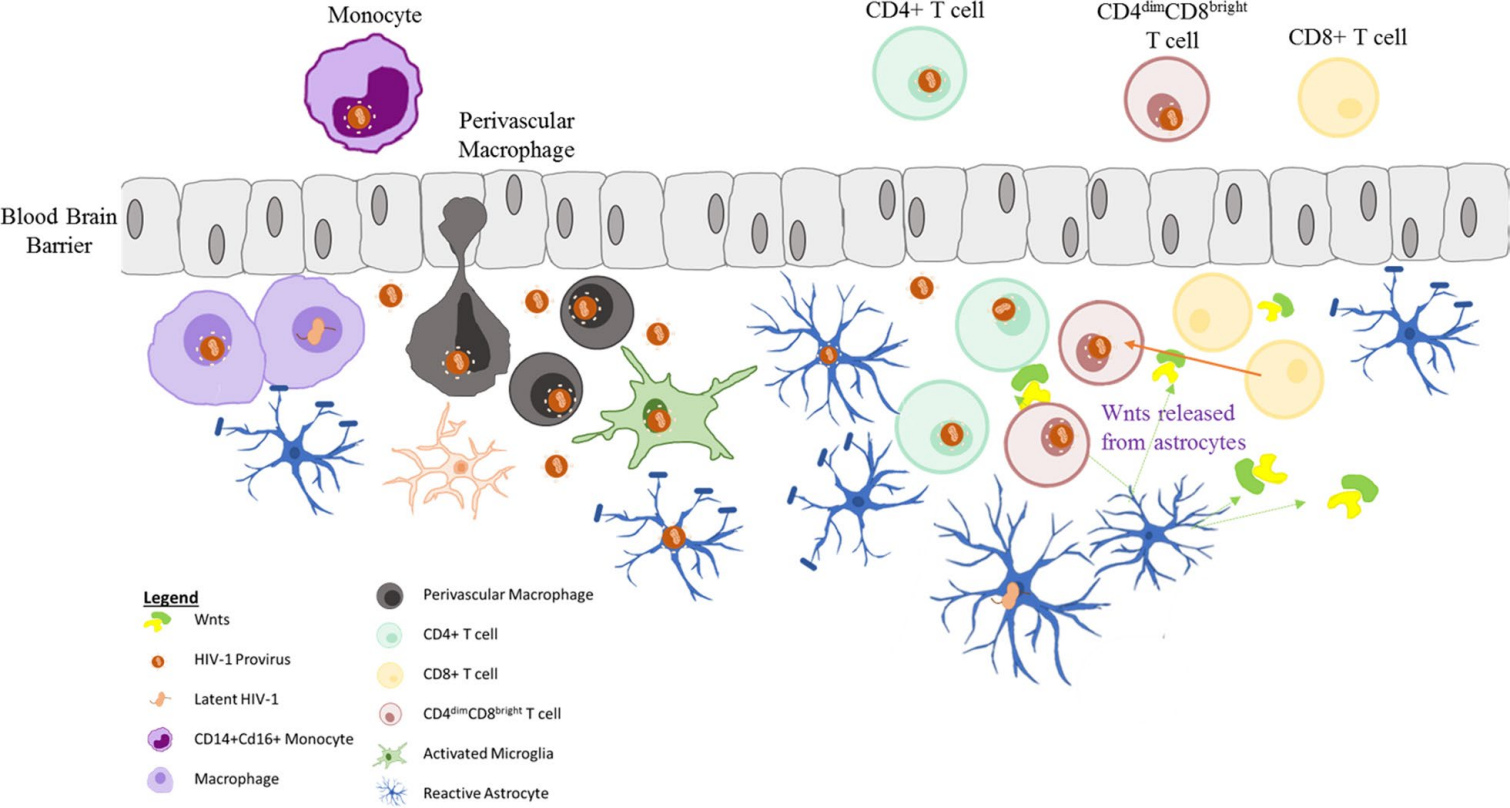
Immune cells-mediating HIV neuroinvasion. To date, four immune cell types are described to support HIV invasion into the CNS. Those include CD14 + CD16 + monocytes, perivascular macrophages, CD4 + T cells, and CD4^dim^CD8^bright^ T cells. CD4^dim^CD8^bright^ T cell may invade the brain as such, or the phenotype is generated in the brain as CD8 + T cells enter the brain and through the Wnt-rich environment in the CNS induce CD4 expression on their surface. These HIV-infected immune cells then released HIV in the brain to support infection of microglia and to a lesser extent, non-classical infection, of astrocytes.

HIV invades the brain within approximately two weeks of infection, as demonstrated by both animal [17–21] and human [22, 23] studies. The consequence of this neuroinvasion is two-fold, induction of inflammatory responses that culminate in the manifestation of HIV-Associated Neurocognitive Disorders (HAND) and the establishment of the brain as a reservoir for HIV. Yet, two decades after identifying HIV as the etiologic agent of AIDS, there is still some controversy regarding which infected immune cells disseminate HIV into the brain. Earlier studies identified monocytes as the trojan horse of HIV, and especially those that are CD14 + CD16 + cells [24–27]. However, monocytes are not *productively* infected by HIV and only when they differentiate to macrophages can they support productive HIV replication [28–30]. Recent studies also demonstrated that while monocytes harbor HIV DNA they also express higher level of an HIV-repressor factor, β-catenin, but loose that expression once they differentiate into macrophages, supporting HIV replication in macrophages [31]. Further, the level of HIV replication in macrophages likely depends on their phenotype (M1/M-2-like) and tissue site. Hence, the paradigm that exists in HIV neuroinvasion is that monocytes harbor HIV DNA, migrate to the CNS, differentiate into macrophages allowing dissemination of HIV into the brain. Emerging evidence supports a role for CD4 + T cells in HIV neuroinvasion [32–34], especially since there is a greater appreciation that the brain is not an immune privileged site [35]. T cells home into the brain at low numbers to survey the brain and in response to neuroinflammation they home at greater numbers, although with slower kinetics. There is also a greater appreciation for a lymphatic system in the brain which was first described in the mid-1940s and re-evaluated using sophisticated studies led by the Kipnis group [36–38]. These observations then provide a rationale by which infected CD4 + T cells can disseminate HIV in the brain. Indeed, CD4 + T cells are detected in the brain of SIV-infected macaques [39, 40] and in human CSF [41]. Further, HIV-infected CD4+ T cells migrated into the CNS in humanized mice [17]. Despite these studies, the presence of infected CD4 + T cells in the brain parenchyma of deceased HIV + individuals are not welldocumented. On the other hand, CD8 +T cells are found. Modeling the role of lymphocytes in HIV neuroinvasion in humanized mice, a recent study found that infected CD4 + T cells can home to the brain but die fairly quickly, which can potentially explain the inability to find infected CD4 + T cells from human post mortem brains [17]. Of interest, is a recent observation that a unique population of CD8 + T cells which express CD4 on its surface, called CD4^dim^CD8^bright^ T cells mediates HIV neuroinvasion [17, 42–47]. CD4^dim^CD8^bright^ T cells constitute ~ 3–5% of total CD8 + T cells among healthy and HIV + chronically infected patients and up to 15% of CD8 + T cells from HIV infected long-term non-progressors [43]. Blood CD4^dim^CD8^bright^ T cells are highly enriched in anti-HIV responses [43], constituting approximately 60% of the anti-HIV tetramer responses and are polyfunctional and cytolytic [43]. β-catenin, the central mediator of the Wnt/β-catenin pathway, mediates CD4 expression on mature CD8^+^ T cells [46]. Using NOD/SCID/IL-2rcγ-/- mice reconstituted with human PBMCs (NSG-huPBMC), CD4^dim^CD8^bright^ T cells were reported to mediate HIV neuroinvasion yet due to a high level of the antiapoptotic protein, Bcl-XL, they resist HIV-mediated cytopathy [33, 48]. Further the Wnt-rich environment in the CNS induced CD8 single positive T cells to become CD4^dim^CD8^bright^ T cells both in vitro and in vivo[17]. Expression of CD4^dim^CD8^bright^ (not CD8 single positive) T cells was inversely associated with HIV in the brain and brain CD4^dim^CD8^bright^ T cells of HIV-infected NSG-huPMBC mice exert anti-HIV cytolytic activity [17]. These findings highlight a significant role for CD4^dim^CD8^bright^ T cells in HIV neuroinvasion (https://www.nimh.nih.gov/news/research-highlights/2022/t-cells-help-hiv-enter-and-persist-in-the-brain). A model of key cell types, to date, reported to mediate HIV neuroinvasion is illustrated in Fig. 1.

### Mechanisms of CD4-independent HIV infection of non-classical cells

**Fig. 2:**
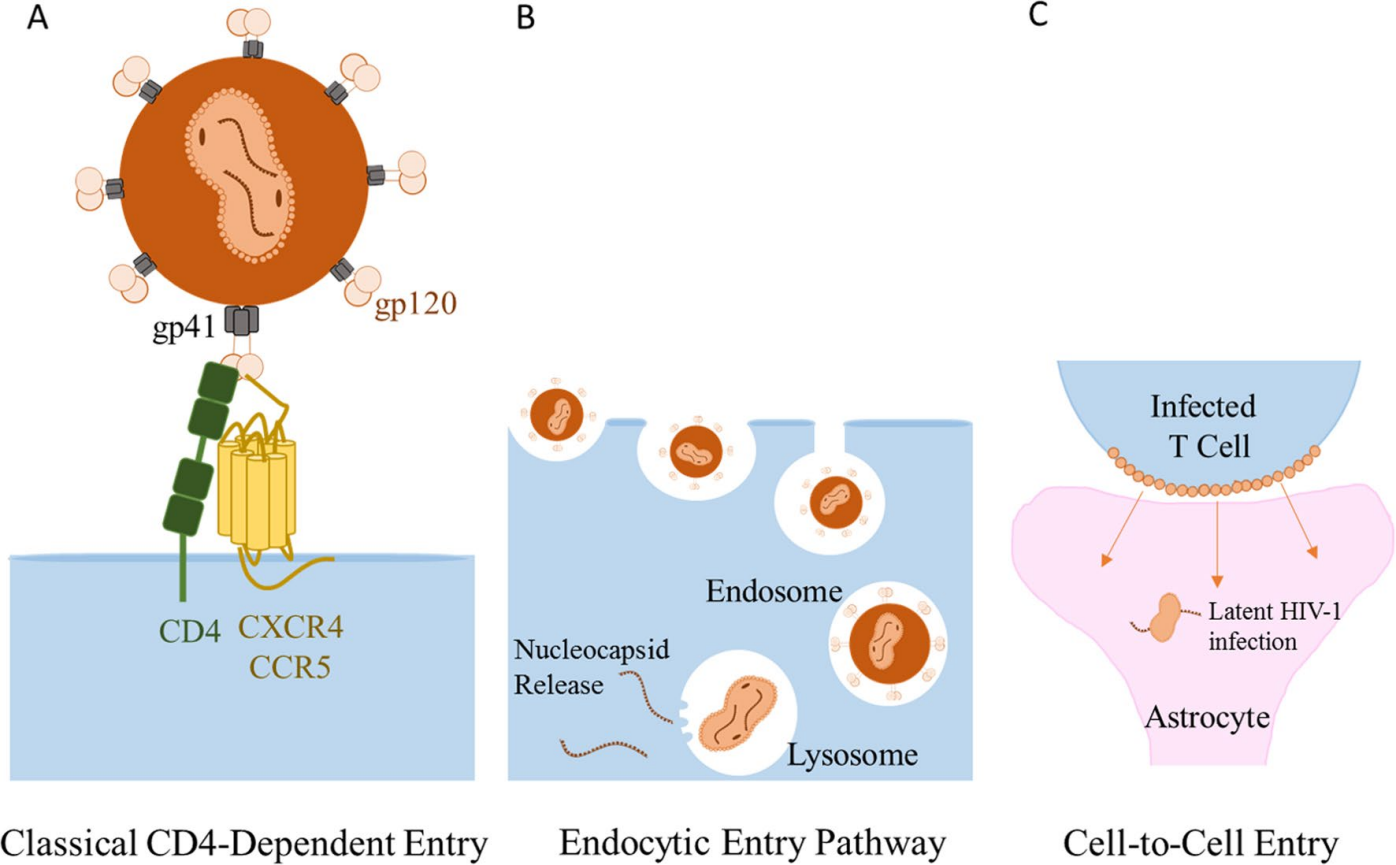
Conventional and unconventional mechanisms of HIV infection of target cells: HIV infection of target cells through gp120/CD4/chemokine receptor interactions is the most efficient means of HIV infection. Alternative mechanisms include cell-to-cell transfer of HIV between infected cell and a target cells and endocytic pathway of viral infection.

The primary and most efficient means by which HIV enters cells is through binding of its envelope (gp120) to the host CD4 molecule (its receptor) leading to a conformational change in both gp120 and CD4 exposing the V3 region of gp120 allowing it to bind to one of the chemokine-coreceptors (CCR5, CXCR4) and for HIV gp41 to mediate fusion with the host cell membrane to release the viral core and initiate the cascade of HIV replication from uncoating, to reverse transcription, to integration, to transcription, to protein synthesis, to assembly, and finally budding [49–51]. However, two alternative mechanism(s) of HIV infection, albeit less efficient, are described, which are cell-to-cell transfer of HIV and endocytosis [52, 53] (Fig. 2). Both of these mechanisms have been described for HIV entry into astrocytes [54–63].

In an elegant study, human astrocytes and HIV-infected lymphocytes were co-cultured and HIV infection from lymphocyte to astrocyte was monitored by sophisticated imaging approaches, which included live-imaging, confocal and three-dimensional electron microscopy [55]. Images show HIV transmitted by cell-to-cell contact from T cells to astrocytes. The images also show two means of contact, one where the infected lymphocytes were folded into astrocytes and another where a virologic synapses was formed between the two cells allowing for the immature budding of HIV particles through filopodial extensions from lymphocytes to astrocytes.

The endocytic pathway is often hijacked by viruses to enter host cells [64]. Although there are four described mechanisms of endocytosis: phagocytosis, micropinocytosis, caveolin-mediated endocytosis, and clathrin-mediated endocytosis, described based on particle size and other markers, caveolin-mediated endocytosis is the most commonly used by viruses, including HIV [65]. Once HIV is within the endosome, its fate can be either secreted as the endosome recycles and releases its content, degraded due to the acidic pH of the endosome as it matures from an endosome to a lysosome, or the virus fuses with the vesicular membranes to release its core to the cytoplasm and undergoes its normal replicative cycle [58, 65]. Factors that can disrupt the maturation of endosome to lysosome, such as methamphetamine which increases pH of endosome or chloroquine, can allow escape of HIV from the endosome to the cytoplasm [63]. While endocytosis is not a highly efficient mechanism of HIV entry, it can promote HIV infection in non-classically (e.g. CD4 negative) target cells [65, 66].

### HIV infection of astrocytes

Astrocytes, depending on their location, constitute 30–70% of the brain and perform vital functions to maintain brain homeostasis, including serving as the food source for the brain through storing glycogen which is then hydrolyzed to ATP and glucose and lactate, they regulate the level of water and other extracellular ions such as potassium and sodium, they secrete neurotrophic factors, and regulate the level of neurotransmitters such as glutamate which in excess is neurotoxic, and their end feet wrap around endothelial cells to maintain the integrity of the blood brain barrier. Astrocytes also have immune modulatory functions including release of cytokines and chemokines as a signal to alert immune cells to sites of brain inflammation/injury, promote myelination and phagocytosis of the ends of damaged neurons, and secret factors that promote neurogenesis. Due to these prolific functions, any disruption in their role can negatively impact the brain. Indeed, over the last several years, there is a greater appreciation for the role of astrocytes in health and disease, where in a number of neurodegenerative diseases, astrocytes either have a loss or gain of function [67]. In HIV, dysregulation of astrocytes is well documented [68, 69].

Astrocytes are CD4 negative [70], express CXCR-4, and depending on their developmental and activation state they may express CCR5. While HIV infection of astrocytes is independent of CD4, several alternative receptors were identified, albeit, still controversial as some are identified in astrocytoma cell lines and not primary human astrocytes. These alternative receptors for HIV entry in astrocytes include an orphan chemokine co-receptor called D6, galactocerebroside, human mannose receptor, and an unidentified molecule [62, 71–73]. In the case of mannose receptor, its binding to gp120 leads to HIV endocytosis with low levels of viral escape from the endocytic compartment, reverse transcription, and integration of HIV [59, 62, 63]. Further, astrocytes are infected by cell-to-cell contact, as described above. Table 1 highlights some of the studies demonstrating HIV infection of astrocytes using primary human astrocytes, astrocytoma cell lines, animal models (mice and macaque), and human brain tissue samples.

**Table 1:** List of some studies in support and absence of HIV infection of astrocytes

Study reference	Year	HIV infection of astrocytes (Yes/No)	Relevance to astrocytic HIV detection
[159]^d^	1987	Y	HIV Ags were detected in Adult AIDS human brains by IHC
[160]^a^	1987	Y	HIV Ags detected from Adult AIDS patients and evidence of virus budding
[161]^a^	1994	Y	HIV Ags and nucleic acids from pediatric AIDS patients using IHC and HIV DNA/RNA in situ hybridization
[162]	1995	Y	HIV mRNAs and proteins detected in HIV + brains, with or without dementia, and HIV mRNAs correlated with dementia
[54]	1995	Y	In vitro studies, human fetal astrocytes infected by free virus and more efficiently in co-cultures with T-tropic infected T cells
[163]	1996	Y/N	Cell free HIV infection is limited and enhanced by co-culture with infected monocytes/macrophages
[164]^c^	1998	Y	In vitro and in vivo studies in macaques. In vitro, adult macaque astrocytes infected by SIVmac251. In vivo, evidence of infection and ex vivo SIV + astrocyte reactivated by cytokines
[61]	1999	Y	In vitro infection of human astrocytes and visualization by microscopy of virions in clathrin-coated pits and cytoplasmic vacuoles
[165]	2000	Y/N	Adult macaque astrocytes infected with SIVmac251 show early transcripts/proteins, restriction not limited by HIV nef,and virus not reactivated by cytokines
[166]	2001	Y/N	Low level of infection determined using SVG-A, a simian virus 40 (SV40)-transformed human astrocyte cell that is overcome by overcoming HIV entry requirement
[94]	2001	Y	Human fetal astrocytes infected with VSV-G pseudotyped virus shows productive infection than lower level over time
[75]^a^	2003	Y	Pediatric and adult human LCM captured astrocytes tested for HIV by IHC and HIV DNA PCR
[88]^c^	2003	Y	Macaque astrocytes show SIV infection in acute and chronic disease by CCR-5 virus
[156]^a^	2004	Y	HIV envelope (V3 region( is distinct) from LCM captured astrocytes than monocytes/,macrophages
[62]	2004	Y	In vitro studies of primary human fetal astrocyte and astrocytoma cell lines demonstrating use of mannose receptor for HIV entry, visualized by transmission electron microscopy,
[167]^a^	2006	Y	LCM captured astrocytes from human brains with varying degrees of HAND, show integrated HIV DNA in all categories of HAND tested and with higher level when adjacent to macrophages
[103]	2007	Y	Progenitor-derived astrocytes demonstrate low level of HIV productive infection
[90]	2011	Y	Low level of HIV in human astrocytes is enhance by IFNγ through blocking of Wnt/signaling
[168]	2011	Y	Low level of infection modeled in human fetal astrocytes and SIV infected macaques
[106]	2012	Y	Low level of transcriptional activity of HIV demonstrated in primary human fetal astrocytes and astrocytoma cell line expressing HIV-LTR, promoter induced by disruption of Wnt/β-catenin signaling
[84]	2014	Y	HIV LTR in human fetal astrocytes is under epigenetic regulation (class I histone deacetylases (HDACs) and a lysine-specific histone methyltransferase, SU(VAR)3–9)
[169]	2014	Y	Rapid infection of HIV then a decline and use of CD81 for HIV entry into astrocytes
[170]	2015	Y	HIV from infected primary human fetal astrocytes is infectious and transmits virus to lymphocytes in vitro
[55]	2015	Y	Visualization of HIV transfer from T cells to astrocytes
[171]	2015	Y	protein kinase C agonist reversing HIV latency in astrocytes
[70]	2017	N	A fusion reporter assay does not show HIV fusion to astrocytes or productive infection
[87]	2018	Y	Non-proliferating astrocytes sustained long term infection of HIV. Latently infected cells were not inducible
[172]	2019	Y	Lately infected human astrocytes
[91]^d^	2019	N	Astrocytes from cART suppressed brains are not positive for HIV DNA measured by DNAscope (caveat to study rebutted elsewhere)
[173]	2019	Y	In vitro HIV infection of human astrocytes, with or without Morphine
[85]^a^^b^	2020	Y	Integrated HIV DNA from maximally suppressed donors shows infection frequency of 0.4–5.2% integrated HIV gag DNA and 2–7% are HIV gag mRNA positive Xenotransplanted HIV infected astrocytes into humanized mice demonstrates HIV replication in astrocytes and ability to transfer virus from brain to other peripheral organs through immune cells
[56]	2020	Y	Productive HIV replication and integrated proviral DNA detected from in vitro infected astrocytes
[174]	2020	Y	A pseudotyped doubly labeled fluorescent reporter red/green (R/G)-HIV-1 shows two patterns of infected astrocytes, those that are restricted and resistant to reactivation, and those that are actively infected and inducible
[175]^a^	2021	Y	Brains from cART suppressed donors show astrocytic integrated HIV DNA. In vitro HIV infected human primary astrocyte show low level of astrocytic HIV integrated DNA and latency without cART
[176]	2021	Y	Latently-infected human astrocytes in vitro
[177]	2022	Y	Generation of adult astrocytic cell line that are HIV-infected demonstrate HIV provirus in adult astrocytes

The list is not intended to be comprehensive and is presented in chronological order. Infection is defined as HIV entry and detection of either latent HIV, low level HIV RNA or protein, or infectious particle release

*Ags* Antigens, *IHC* Immunohistochemistry

^a^Human brain post-mortem studies

^b^Humanized mice

^c^SIV-macaque studies

^d^indicated as no and rebuttal based on author’s own figures were provided in [92]

HIV infection in astrocytes is not robust and is either low-level or latent. An extensive body of literature has demonstrated that astrocytes fulfill classical criteria defining “HIV reservoirs” which include: (1) Harboring provirus: In vitro and in vivo studies indicates that astrocytes harbor integrated HIV DNA (provirus) [74–77]. In fact, 1–3% of astrocytes from HIV infected brains harbor HIV provirus [75, 78]. The size of astrocytic pool of HIV is impressive considering their vast number in the brain and in context of the size of HIV latently infected resting CD4 + T cells, which is quite small constituting approximately 10^5^–10^7^ of CD4 + T cell, with 62-fold higher numbers of CD4 + T cells harboring provirus that is replication competent, yet non-inducible [79, 80]. Further, these calculations do not take into consideration other cellular reservoirs and are probably a vast under estimation of the size of the HIV reservoir in the entire body. Astrocytes in essence could constitute a comparable pool of latent HIV as that defined for resting T cells since there are billions of astrocytes in the human brain. (2) The provirus can be reactivated and is infectious: HIV from infected astrocytes is induced/reactivated by inflammatory signals [81–83]. (3) Detection of low-level HIV transcripts without protein expression: HIV-infected astrocytes express very low levels of early and late transcripts and no detectable protein can be measured. (4) HIV promoter is under epigenetic regulation in astrocytes: class I histone deacetylases (HDACs) and a lysine-specific histone methyltransferase, SU (VAR)3–9, regulate silencing of HIV transcription in astrocytes [84]. (5) Evidence for HIV integrated DNA in post-mortem tissue described by a number of groups (Table 1) and more recently in [85].

HIV in latently infected astrocytes is responsive to latency reversing agents [84, 86], although just as was described for resting CD4 + T cells, there is integrated HIV that is not responsive to latency reversing agents and yet are replication competent [79, 87]. Further, HIV DNA and protein were detected in SIV infected macaques [88, 89]. The rate of integrated HIV DNA in astrocytes, based on in vitro [84, 90] and ex vivo studies (post-mortem tissue from HIV infected individuals) [74, 78] is approximately 1–3%. Other studies have shown that the percentage of infected astrocytes correlates with their proximity to myeloid cells, with up to 20% of astrocytes positive for HIV DNA when in close proximity to infected macrophages among donors who had dementia/HIV encephalitis, [74], however, this may be an over estimate as a result of astrocytes engulfing HIV-infected macrophages [70] or potential contamination of astrocytes with macrophages. Nonetheless, in captured astrocytes from postmortem brain of HIV-infected individuals without dementia/encephalitis, whereby the astrocytes were distant from microglia or perivascular macrophages, HIV in astrocytes was close to 1 and 6% from individuals with both dementia and advanced encephalitis [74]. This is consistent with recent data using advanced technology such as RNA and DNAscope in combination with laser capture of astrocytes from human brains [85]. Given the sheer number of astrocytes in the human brain, 1% of infected astrocytes can far exceed the resting memory T cell reservoir pool. The question remains whether astrocytes can harbor replication competent HIV in vivo. A recent study modeled this in vivo using NOD/scid-IL-2Rgc null (NSG) mice xenotransplanted with human astrocytes and reconstituted with human peripheral blood mononuclear cells (huAstro/HuPBMCs). The model targeted a specific question and by design was reductionist in nature, and it demonstrated that infected astrocytes harbor replication competent HIV and release it to periphery [85]. The authors demonstrated that astrocytes support HIV infection in vivo and egress to peripheral organs, at least in part, through trafficking of infected CD4 + T cells out of the brain [85].

Despite these extensive studies demonstrating low level of HIV replication and/or latency in astrocytes (Table 1), there is still a level of skepticism regarding HIV infection of astrocytes. Two studies in particular are in opposition of the majority of published studies [70, 91]. Ko et al. [91] analyzed post-mortem tissue from HIV + donors for HIV presence using DNA and/or RNA scope. Specifically, using RNA- and DNAscope with immunohistochemistry to detect HIV DNA and RNA in macrophages (defined as CD68 + or CD206 +) and astrocytes (defined as GFAP +) from post-mortem brains of individuals with undetectable plasma viral load, they concluded that astrocytes do not harbor HIV in these samples [91]. There were several technical concerns that negate the conclusion that astrocytes do not harbor HIV in vivo, which were rebutted in a letter to editor [92] and responded to [93]. Most significantly, the title and conclusion of this study is rather bold and affirmative that HIV is found in macrophages only and not astrocytes, which is problematic because this is based on limited donors, limited regions of the brain, and most critically astrocytes are heterogenous and they were defined by GFAP expression only and did not include other markers such as S100β. Indeed, a caveat that plagues the field is the heterogeneity of astrocytes which should not be defined by GFAP expression only and as the data are based on GFAP + it is a far reach to conclude the lack of infection of all astrocytes as no other markers were used. The study by Sattentau et al. employed luciferase and green fluorescent protein (GFP) reporter viruses and cell fusion and imaging assays and concluded that HIV does not infect human fetal astrocytes [70]. This assay, although elegant, has its limitation as it may not detect signal from a small fraction of cells and the assay does not measure HIV integration, which likely occurs through non-classical means, as well as culturing conditions and developmental stage of HFAs may be a factor in their ability to be infected, albeit, a restricted infection.

### Overcoming restricted and/or latent HIV infection in astrocytes

Astrocytes infected with VSV-G pseudotyped virus to bypass receptor entry recruitment, demonstrated that there is no intrinsic restriction to HIV release [94], yet in comparison to virus output from VSVg-G pseudotyped infected HeLa cells, the level is much lower. This suggests that there are intrinsic host factors that suppress robust HIV replication in astrocytes. Several were identified which include low constitutive expression of an essential protein for Rev function known as Sam68 [95–97], enhanced double-stranded (ds) RNA-activated protein kinase (PKR) associated with a block in protein synthesis of TAR RNA binding protein (TRBP) [98–101], endogenous expression of the downstream effector of the Wnt signaling pathway, T cell factor 4 (TCF-4) that represses HIV LTR activation [102] and replication [103], and abundance of Beclin1, an autophagic protein [104]. This restricted replication can be overcome by certain signals such as cytokines by suppressing the repressor as in the case of IFNγ, GM-CSF, or even Methamphetamine which inhibit β-catenin signaling to overcome TCF-4 mediated suppression of HIV transcription [90, 103, 105–107] or RNA interference of Beclin [104]. As such, these conditions may explain the controversial nature of whether astrocytes are infected or not as the microenvironment, activation state, and even developmental stage of astrocytes may dictate their extent of permissiveness to HIV infection, replication, latency, and/or reactivation.

### HIV infection of Microglia

Soon after neurological impairments were described in HIV/AIDS patients, analyses of brain tissue obtained post-mortem from pediatric and adult patients that succumbed to AIDS demonstrated the presence of HIV protein and nucleic acid in the brain. Further studies revealed the presence of HIV-infected macrophages/microglia in brain tissue via immunohistochemistry, in situ hybridization, and laser capture microdissection followed by PCR [75, 78, 91, 108–114]. Brain macrophages and microglia express similar surface markers (e.g. CD68, CD14, and CD45) and therefore, it can be difficult to differentiate microglia from macrophages. However, based on location and morphology one study estimated that microglia account for at least two-thirds of HIV-infected cells in the brain of people with HIV encephalitis [108].

Microglia express low levels of CD4 and the HIV co-receptor CCR5 [115, 116]. Viruses detected in the CNS of PLWH are predominately CCR5-tropic and many infect cells expressing low levels of CD4 (macrophage-tropic or M-tropic) [117]. Accordingly, multiple CCR5-tropic M-tropic HIV strains have been shown to infect primary microglia in vitro [115, 118–122]. HIV infection of microglia is not restricted despite high expression levels of the sterile alpha motif and histidine/aspartic acid domain-containing protein 1 (SAMHD1), a deoxynucleoside triphosphohydrolase (dNTPase) that can restrict HIV infection by reducing cellular dNTP pools [123]. This is attributed to microglia residing in a G1-like state which promotes upregulation of cyclin kinase 1 resulting in phosphorylation and inactivation of SAMHD1 [124].

HIV infection of microglia results in the production of viral proteins and the release proinflammatory cytokines/chemokines, reactive oxygen species (ROS), and reactive nitrogen species (RNS) creating an inflammatory environment resulting in activation of bystander microglia and astrocytes [125–128]. Viral proteins like gp120, Tat, and Vpr can directly or indirectly mediate neuronal cell injury resulting in neuronal cell damage and loss which may contribute to the development of HAND [126, 128]. HIV gp120 and Tat also stimulate microglia to release proinflammatory cytokines TFN-α and IL-1β [129, 130]. Elevated levels of TFN-α and IL-1β have been observed in the CSF of PLWH with HAD [131]. TNF-α is directly toxic to neurons in vitro and both TNF-α and IL-1β can facilitate neuronal injury by increasing the release of neurotoxic molecules (e.g. ceramide, L-cysteine) [125, 129, 130, 132, 133].

CNS inflammation persists in PLWH receiving cART [2, 8, 10], suggesting the continued presence of HIV-infected cells in the brain. Indeed, HIV DNA + cells have been detected in the CSF of aviremic ART-suppressed PLWH [134]. HIV DNA + and HIV RNA + cells have also been observed in postmortem brain samples of individuals with sustained cART suppression that died of unrelated causes [91, 109]. Microglia are thought to represent a key cellular reservoir of HIV in the brain under ART as they are long-lived, slowly self-renew, and are resistant to HIV-induced apoptosis [135–137]. In SIV-infected, ART suppressed rhesus macaques, latently-infected macrophages/microglia have been isolated from the brain [138, 139]. Latent HIV infection has also been demonstrated in iPSC-derived microglia and immortalized human microglia cell line models [140–147].

Whereas there is vast information regarding HIV latency in CD4 + T cells, particularly in peripheral blood CD4 + T cells, there is much less knowledge regarding key aspects of HIV latency in microglia. HIV latency is established in CD4 + T cells when activated infected CD4 + T cells transition to resting memory cells resulting in transcriptional silencing of the virus [148]. Multiple mechanisms are involved in maintaining HIV latency in CD4 + T cells including epigenetic modifications of chromatin and the levels of host transcriptional activators and repressors [148]. Microglia cannot be readily obtained from PLWH to study the mechanisms of HIV latency. In cell line models, Coup-TF interacting protein 2 (CTIP2) has been identified as an important regulator of HIV transcription in human microglia [145–147]. CTIP2 promotes latency by recruiting HDAC1, HDAC2, and HMT Suv39H1 to the HIV promoter resulting in epigenetic changes and the formation of heterochromatin [145–147]. Host transcriptional factor nuclear receptor related 1 (Nurr1) has also been shown to induce silencing of HIV transcription in an immortalized microglia cell line model. Nurr1 binds directly to the HIV LTR and recruits CoREST transcription repressor complexes (CoREST, HDAC1, G9a, and EZH2) resulting in chromatin remodeling [142]. Activation of the glucocorticoid receptor, which directly associates with the HIV LTR, has also been shown to promote HIV latency in a microglia cell line model [143]. Interestingly, co-culture with healthy GABAergic cortical and dopaminergic neurons (but not motor neurons) has also been shown to promote HIV latency in a human microglia cell line model and in iPSC-derived microglia [140]. GABAergic cortical and dopaminergic neurons produce glucocorticoids while motor neurons do not, further supporting a role for the glucocorticoid receptor in regulating HIV latency in microglia [140].

Future studies in SIV/NHP and HIV/humanized mouse models may provide further insight into the establishment of HIV latency in microglia and the maintenance of latently infected cells. Sequence analysis of near-full-length genome proviruses isolated from T cells of ART-treated PLWH suggest that clonal proliferation of CD4 + TH_1_ cells drives stabilization of the HIV reservoir in T cells [149]. However, the role of clonal expansion in maintaining the HIV reservoir in microglia in vivo is not known. It will also be important to determine the relative contribution of microglia to the HIV reservoir and to virus rebound during therapy interruption.

### HIV compartmentalization within resident brain cells

There is clear evidence for genetic evolution of HIV compartmentalization in the brain that is different from that of lymphoid tissue [23, 117, 150–152]. HIV in CSF had a unique half-life compared to blood among a subset of patients who have evolved M-tropic HIV in the CNS [153]. Characterization of HIV quasispecies in CSF revealed that compartmentalization of HIV in the CNS is associated with HAND, implicating a role for HIV genetics in the development of neuropathology [154]. Early studies investigating CNS strains in CSF and brain parenchyma found distinct envelope, LTR, and other genes [155], however, new virologic techniques developed to unambiguously and physiologically determine HIV genotypes and phenotypes have not been applied to CNS HIV. Limited studies evaluated the extent of HIV compartmentalization within resident brain cells (astrocytes vs. macrophages/microglia). To date, a small study based on brain tissue from two HIV-E patients found evidence for HIV compartmentalization in astrocytes that is distinct from macrophages/microglia [156]. There is a need for more detailed studies, especially those evaluating HIV genetic evolution in era of cART and in relation to various clinical stages of HAND.

## Conclusions

Elimination of the persistent reservoir of latently-infected cells is the greatest challenge to HIV eradication. Latently-infected CD4 + T cells are considered the most important HIV reservoir and obstacle to an HIV cure [157, 158]. However, prior research has also shown that resident glial cells in the brain are infected by HIV and may establish latency [81–83, 140, 143, 145–147]. Therefore, elimination of the T cell reservoir alone may not eradicate HIV and HIV cure approaches may be needed that penetrate the brain and target alternative cell types like glial cells. Regardless of whether HIV establishes true latency in glial cells, HIV-infection persist in the brain of cART-suppressed PLWH and contributes to neuroinflammation and dysfunction. Therefore, future studies are needed to better understand the mechanisms of HIV persistence in glial cells in vivo and their relative contribution to the HIV reservoir of virus rebound.

## Data Availability

Not applicable.

## Abbreviations

AIDS: Acquired immunodeficiency syndrome
ANI: Asymptomatic neurocognitive impairment
ATP: Adenosine triphosphate
cART: Combination antiretroviral therapy
CNS: Central nervous system
CoREST: REST co-repressor 1
CSF: Cerebral spinal fluid
CTIP2: Coup-TF interacting protein 2
DNA: Deoxyribonucleic acid
dNTPase: Deoxynucleoside triphosphohydrolase
DS: Double stranded
GFAP: Glial fibrillary acidic protein
HAD: HIV-associated dementia
HAND: HIV-associated neurocognitive disorders
HDAC: Histone deacetylase
HIV: Human immunodeficiency virus
LTR: Long terminal repeat
MND: Mild neurocognitive disorder
NSG: NOD/scid-IL-2Rgc null
Nurr1: Nuclear receptor related 1
PCR: Polymerase chain reaction
PKR: RNA activated protein kinase
PLWH: People living with HIV
ROS: Reactive oxygen species
RNA: Ribonucleic acid
RNS: Reactive nitrogen species
PBMC: Peripheral blood mononuclear cell
SAMHD1: Sterile alpha motif and histidine/aspartic acid domain containing protein 1
SIV: Simian immunodeficiency virus
TCF-4: T cell factor 4
TRBP: TAR DNA binding protein
VSV: Vesicular stomatitis virus
